# Dose Escalation and Healthcare Resource Use among Ulcerative Colitis Patients Treated with Adalimumab in English Hospitals: An Analysis of Real-World Data

**DOI:** 10.1371/journal.pone.0149692

**Published:** 2016-02-26

**Authors:** Christopher M. Black, Eric Yu, Eilish McCann, Sumesh Kachroo

**Affiliations:** 1 Merck & Co, Inc., Kenilworth, United States of America; 2 IMS Health Ltd, London, United Kingdom; 3 Merck Sharp & Dohme Ltd, Hoddesdon, United Kingdom; Corvinus University of Budapest, HUNGARY

## Abstract

**Objective:**

To describe the real-world use of adalimumab for maintenance treatment of ulcerative colitis (UC) and associated healthcare costs in English hospitals.

**Design:**

Retrospective cohort study.

**Setting:**

Analysis of NHS Hospital Episode Statistics linked with pharmacy dispensing data in English hospitals.

**Patients:**

Adult UC patients receiving ≥240mg during adalimumab treatment induction, subsequently maintained on adalimumab.

**Outcomes:**

Frequency and pattern of adalimumab use and dose escalation during maintenance treatment and associated healthcare costs (prescriptions and hospital visits).

**Results:**

191 UC patients completed adalimumab treatment induction. 83 (43.46%) dose escalated during maintenance treatment by ≥100% (equivalent to weekly dosing) (median time to dose escalation: 139 days). 56 patients (67.47%) subsequently de-escalated by ≥50% (median time to dose de-escalation: 21 days). Mean all-cause healthcare costs for all patients ≤12 months of index were £13,892. Dose escalators incurred greater mean healthcare costs than non-escalators ≤12 months of index (£14,596 vs. £13,351). Prescriptions accounted for 96.49% of UC-related healthcare costs (£11,090 of £11,494 in all patients).

**Conclusions:**

Within the cohort, 43.46% of UC patients escalated their adalimumab dose by ≥100% and incurred greater costs than non-escalators. The apparent underestimation of adalimumab dose escalation in previous studies may have resulted in underestimated costs in healthcare systems.

## Introduction

Tumour necrosis factor-alpha (TNF-α) inhibitors infliximab, adalimumab, and golimumab are approved for the treatment of moderately to severely active ulcerative colitis (UC) in adults who have inadequate response, intolerance or contraindications to conventional therapy,[1–3]. The prevalence of adults with UC in England is estimated at 189 per 100,000 population, with 11.5% eligible for treatment with TNF-α inhibitors.[4] The National Institute for Health and Care Excellence (NICE) in England recommends that the choice of TNF-α inhibitor should take patient preference, therapeutic need, adherence, and cost into consideration when prescribing these treatments.[5]

The efficacy of adalimumab in UC was demonstrated in two Phase 3 studies (ULTRA 1 and 2) and an extension study (ULTRA 3).[6–8] The induction regimen for adult UC patients is 160mg at Week 0 and 80mg at Week 2. After induction, the recommended maintenance dose is 40mg fortnightly.[3] The Summary of Product Characteristics (SPC) states that adalimumab should be discontinued if there is no response in 2–8 weeks,[3] and NICE recommends a treatment review after 12 months.[5] Patients with diminishing response may benefit from increasing the dosing frequency to 40mg every week.[3] In ULTRA 2, approximately 16% of previously TNF-α inhibitor-naïve patients were dose escalated from fortnightly to weekly treatment to maintain response or remission.[4]

Drug costs for a maintenance cycle of TNF-α inhibitors (26 weeks) are approximately £4,900–£5,500.[4] An increased dose can translate into higher healthcare costs and may reflect poor disease management. As these factors may influence the choice of agent under current guidance, understanding dose escalation and costs is important from both payer and societal perspectives.

Real-world studies profiling the efficacy of adalimumab exist [9], while studies on dose escalation during maintenance therapy are generally examined in trials which may not reflect actual practice. [7,8,10, 11] NICE and the UK Inflammatory Bowel Disease Audit have used real-world data to estimate that 5–50% of UC patients may require dose escalation.[4,5,8,10–14] This broad figure indicates that further evidence is required to understand adalimumab dosing in clinical practice to assess the burden on payers which may have been previously underestimated.

The objectives of this study were to estimate the proportion of UC patients administered adalimumab who dose escalate (doubling dose or increasing dose frequency [≥100%]), the time to dose escalation and any subsequent de-escalation, and the proportion who de-escalate.

## Methods

### Study Design

**Fig 1:**
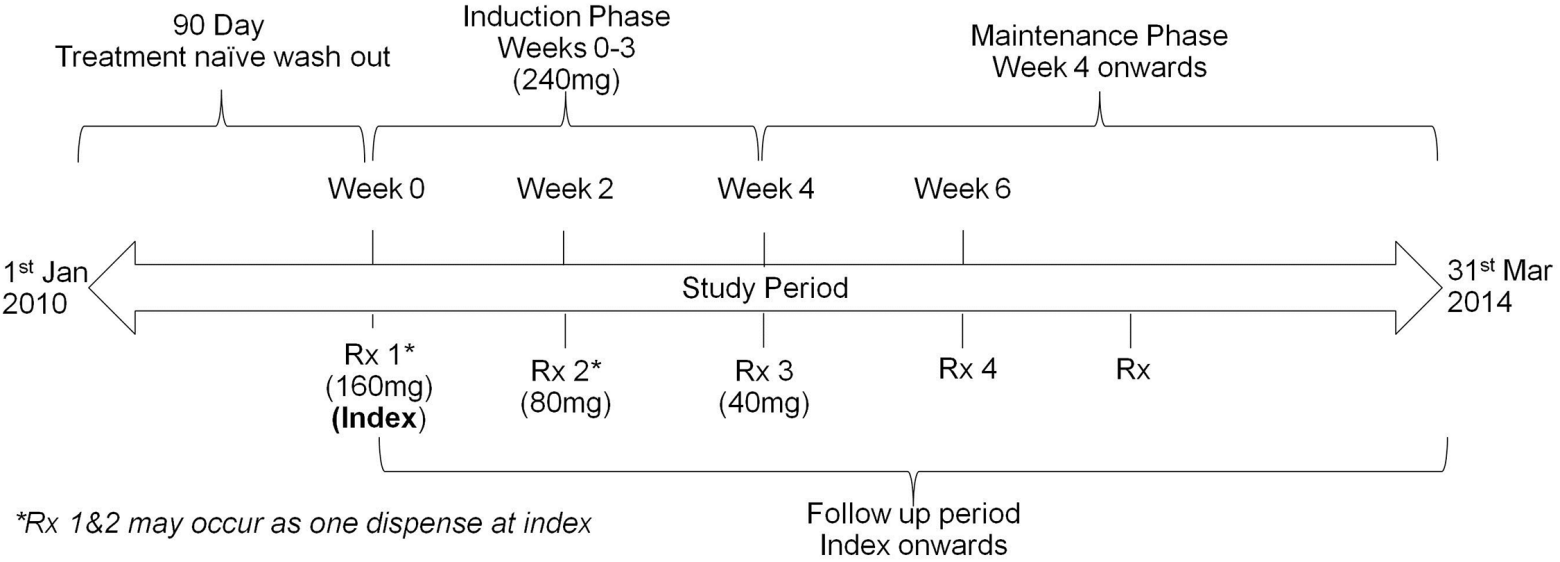
Study design.

This was a retrospective study of UC patients treated with adalimumab between 1 January 2010 and 31 March 2014 in England (Fig 1).

### Data Sources

Data were obtained from the Hospital Treatment Insights (HTI) database (IMS Health Ltd, UK), which links Hospital Pharmacy Audit (HPA) (IMS Health Ltd, UK) and NHS Hospital Episode Statistics (HES) data at the patient transaction level. 3.3 million of all (24.81%) patients in England have HES linked to HPA records and are included in HTI, allowing the linkage of drug and event data. All patient data were anonymized and de-identified prior to analysis. Ethics approval was received by the Independent Ethics and Scientific Advisory Comittee (ISEAC).

### Study Population and Patient Selection

The primary exposure of interest was a prescription for adalimumab during the study period. The date of the first prescription for adalimumab served as the index date (with no exposure to adalimumab 90 days prior to the index date) and was the start of the post-index period where patients were followed-up for the remainder their record in the HTI database.

Patients qualified for inclusion if they were dispensed at least one dose of adalimumab during the study period, had at least one confirmed ICD-10 diagnosis code for UC (K51.0-K51.5, K51.8, K51.9), and were aged ≥18 years at index.

Patients were analysed according to the indicated regimen of adalimumab. The recommended adalimumab induction regimen is 160mg at Week 0 (administered as four injections in one day or two injections per day for two consecutive days [4 x 40mg vials]) and 80mg at Week 2 (2 x 40mg vials). After completion of the induction phase, the recommended maintenance dose is 40mg fortnightly from Week 4 onwards.[3]

Dose escalation was defined as either:

- Fortnightly dispensing at least 100% greater than the initial maintenance dose, or
- Increased frequency of the same maintenance dosage (i.e. from fortnightly to weekly–S1 Fig).

Time to the first dose escalation was measured from the index date. If the days’ supply was missing, a value was imputed by dividing the total dose prescribed by the recommended fortnightly 40mg dose in the SPC.

Only the first dose escalation was assessed. Dose de-escalation was the first subsequent fortnightly adalimumab dose that was ≥50% lower than the escalated dose, back to the recommended maintenance dose. A sensitivity analysis was conducted to capture patients with ≥30% and ≥50% increases in fortnightly adalimumab dose.

Patients were then grouped into three cohorts: patients with a ≥100% dose escalation without subsequent de-escalation (Cohort 1); patients with a ≥100% dose escalation and subsequent ≥50% dose de-escalation (Cohort 2); and patients who did not change their maintenance dose (Cohort 3). Cohorts 1 and 2 were combined for some cost analyses as these were considered similar and both experienced escalated dosing.

### Baseline medications

Medications dispensed to patients at baseline were determined and are summarised in Table 1.

**Table 1:** Baseline patient characteristics.

Characteristic	All patients (n = 191)	Cohort 1 (n = 27)	Cohort 2 (n = 56)	Cohort 3 (n = 108)
Age, mean (SD)	41.0 (14.8)	37.2 (15.4)	42.0 (16.0)	41.4 (14.0)
Male, n (%)	82 (42.9)	11 (40.7)	24 (42.9)	47 (43.5)
Female, n (%)	109 (57.1)	16 (59.3)	32 (57.1)	61 (56.5)
**Year of initial treatment, n (%)**				
2010	22 (11.6)	-	8 (14.3)	14 (13.0)
2011	43 (22.8)	-	23 (41.1)	20 (18.5)
2012	60 (31.7)	7 (28.0)	18 (32.1)	35 (32.4)
2013	64 (33.9)	18 (72.0)	7 (12.5)	39 (36.1)
**Baseline UC medications, n (%)**				
Infliximab	36 (18.8)	7 (29.5)	10 (17.9)	19 (17.6)
Azathioprine	13 (7.0)	6 (22%)	-	7 (6.5)
Glucocorticoids	63 (33.5)	13 (48.1)	24 (42.9)	27 (25.0)
Mesalazine	20 (10.8)	-	7 (12.5)	13 (12.0)

SD, standard deviation; UC, ulcerative colitis. Cohort 1: escalated ≥100% without subsequent de-escalation; Cohort 2: escalated ≥100% and subsequent de-escalation; Cohort 3: no escalation

### All-cause and UC-related healthcare utilisation costs

All-cause healthcare utilisation was defined as any hospital and pharmacy services used during the 12 months post-index period. UC-related utilisation was defined as all services with a UC diagnosis or related to UC treatment and are a component of all-cause costs. Utilisation and cost categories included hospital outpatient visits, inpatient visits, Accident & Emergency (A&E) visits, and prescriptions. Healthcare utilisation costs were derived from HPA cost data and the Payment by Results in the NHS tariff (2013–2014) [15] and drug costs were evaluated using HPA data for January 2013-December 2014.

### Statistical Analyses

Descriptive statistics were reported for the study outcomes for all patients and for each of the dosing cohorts. Univariate analyses assessed the association of baseline characteristics with cohorts and also patient characteristics with healthcare costs. A bivariate comparison of baseline characteristics between cohorts was performed using Chi-square or Fisher’s exact test for categorical variable, and Kruskal-Wallis rank sum test for continuous variables. Analyses were performed using SAS Version 9.1 (SAS Institute Inc., USA).

## Results

### Study Population

**Fig 2:**
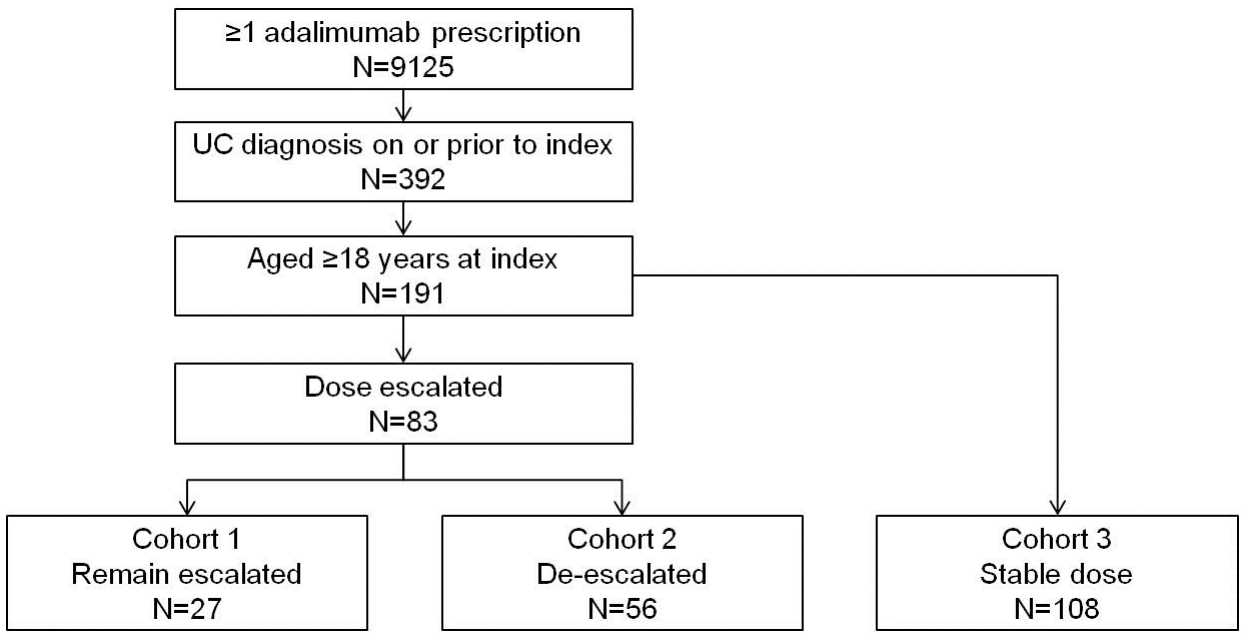
Application of inclusion criteria.

392 patients were identified in the HTI database according to the inclusion criteria (Fig 2). Of these, 201 patients were excluded: 113 had received <240mg of adalimumab during the induction phase and the remaining 88 had insufficient dispensing data in the maintenance phase.

The final study population of 191 patients were grouped into three dosing cohorts: 27 patients with ≥100% dose escalation without subsequent de-escalation (Cohort 1); 56 patients with ≥100% dose escalation and subsequent ≥50% de-escalation (Cohort 2); and 108 patients who did not escalate their maintenance dose (Cohort 3).

#### Patient characteristics and demographics

Patient demographics are reported in Table 1. The mean age was 41 years and 43% of patients were male. A third of patients used corticosteroids prior to starting adalimumab treatment and 18.85% had received prior treatment with infliximab. The median induction phase dose was 320mg.

### Dose escalation during maintenance treatment

83 of 191 patients (43.46%) dose escalated by ≥100% during the maintenance phase within a median time of 139 days. The median fortnightly dose after ≥100% escalation was 107mg. Of those patients who dose escalated by ≥100%, 27 (14.14% of study population/32.53% of dose escalators) remained dose escalated (Cohort 1), while 56 (29.32% of study population/67.47% of dose escalators) subsequently de-escalated by ≥50% (Cohort 2). The median time to dose de-escalation was 21 days. A total of 108 patients (56.54% of study population) did not dose escalate during the post-index period (Cohort 3). Sensitivity analysis showed that 105 patients (54.97%) dose escalated by ≥50% (median time to escalation: 123 days) and 120 patients (62.83%) dose escalated by ≥30% (median time to escalation: 121 days).

The median duration of adalimumab treatment in all study patients was 1.1 years from the index date. Patients who dose escalated had greater median treatment duration (1.4 years) than non-escalating patients (0.9 years).

Those who dose escalated (Cohorts 1 and 2) were significantly more likely to be using glucocorticoids at baseline than non-escalators (p = 0.032 and p = 0.022, respectively). 17 (20.48%) patients who dose escalated were also treated previously with infliximab.

### Healthcare costs in 12 months post-index

**Fig 3:**
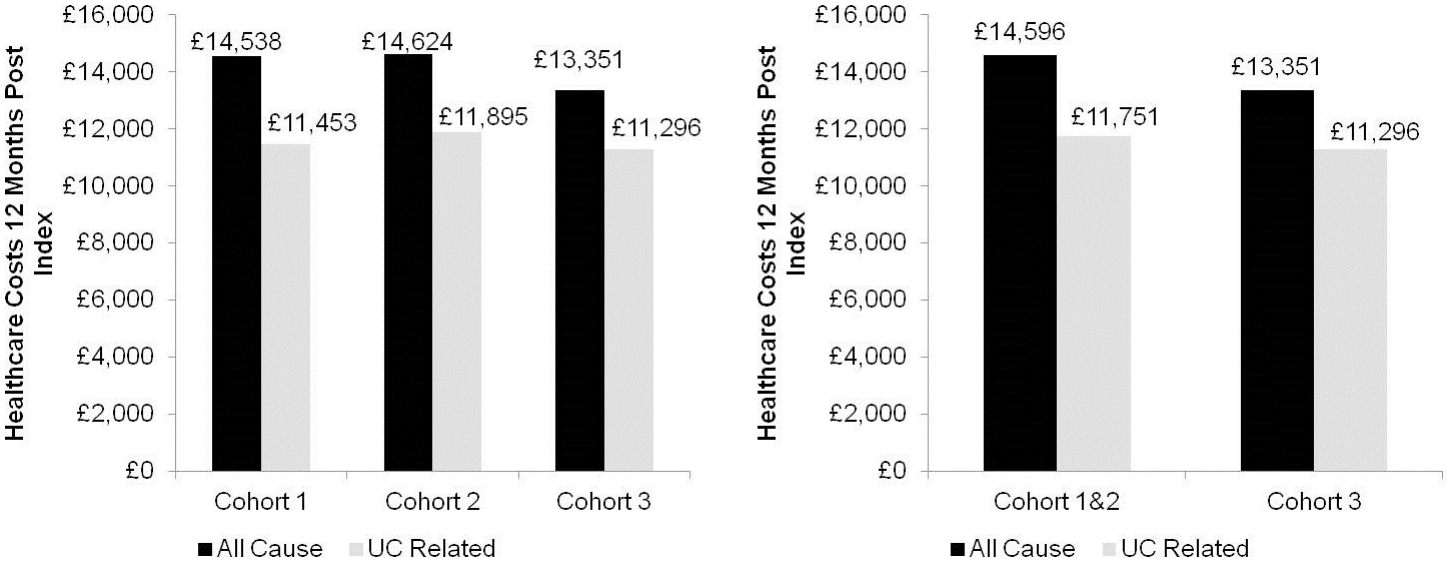
Mean all-cause and UC-related healthcare costs within 12 months post-index.

All-cause and UC-related healthcare costs within 12 months post-index for the study population are shown in Fig 3. Mean UC-related healthcare costs for all patients were £11,494, of which 96.49% was attributable to prescription costs (£11,090). Mean all-cause healthcare costs were £13,892 and 81.23% of costs were attributable to prescriptions (£11,285).

All-cause healthcare costs were significantly higher in females than males (mean difference: £1,710, 95% confidence interval £186-£3,233; p = 0.0281) but this difference was not sustained when limited to UC-related healthcare costs. There were no significant differences related to patient age or year of treatment (data not shown).

Healthcare costs associated with dose escalated patients in 12 months post-index

Mean all-cause healthcare costs within 12 months post-index were £1,245 greater for escalated (Cohorts 1&2) than non-escalated patients (Cohort 3) (£14,596 vs. £13,351) (Fig 3). Similarly, patients who dose escalated had greater mean UC-related healthcare costs than non-escalators (£11,751 vs. £11,296) (Fig 3). Prescription costs accounted for the majority of UC-related healthcare costs for both dose escalated patients (£11,423; 97.21% of UC-related healthcare costs) and non-escalated patients (£10,834; 95.91% of UC-related healthcare costs).

Dose escalation and healthcare costs in prior infliximab users during 12 months post-index

36 of the 191 patients were treated with infliximab prior to index. Of these, 17 (47.22% of prior infliximab users/20.48% of dose escalators/8.90% of study population) dose escalated.

Prior infliximab users who escalated their adalimumab dose had lower mean all-cause (£15,142) and UC-related (£10,537) healthcare costs than non-escalating prior infliximab users (£16,548 and £12,620, respectively).

UC-related healthcare costs in prior infliximab users accounted for 69.59% of mean all-cause healthcare costs in dose escalators (£10,537 of £15,142), compared with 76.264% in non-escalators (£12,620 of £16,548).

## Discussion

This analysis of real-world data provides new insight into adalimumab dose escalation and associated healthcare costs during maintenance treatment for moderately to severely active UC in clinical practice in English hospitals. In our adalimumab-naive study population, 83 (43.46%) dose escalated during the maintenance phase by ≥100%, equivalent to a dosing regimen of ≥40mg weekly or 80mg fortnightly. Of these patients, 56 (29.32%) subsequently de-escalated by at least 50%. When considering patients with dose escalations less than double during the maintenance phase, 55% and 63% of patients escalated by at least 50% and 30%, respectively. Our findings are similar to other real-world data studies including a German UC study reporting dose escalation in 46% of study patients [9] and a Belgian Crohn’s study reporting escalation in 34% of patients [16].

These real-world findings suggest that clinical studies such as ULTRA 3 may have underestimated dose escalation in adalimumab-treated patients as escalation was reported in only 20.41% (120/588) of trial patients. [8]. Furthermore, NICE considered that 16% of patients escalated their adalimumab dose from fortnightly to weekly when developing guidance on the cost-effectiveness of adalimumab in UC [4]. This comparative underestimation may impact their modelling of costs as it has poor generalisability to the English population. The disparity between clinical and real world data studies suggests that trial conditions do not accurately reflect the management needs of European UC patients which will ultimately impact patients’ quality of treatment and healthcare resource use.

Dose escalation is also evident in patients with prior exposure to other TNF-α inhibitors. In our study, one-fifth (18.85%) of patients had received prior treatment with infliximab, and 47.22% of this group went on to escalate adalimumab. The rate of dose escalation in treatment-experienced patients is similar to that experienced by naive patients in this study. This agrees with previous findings in the ULTRA 2 study suggesting that prior TNF-α inhibitor use was a predictor of escalation to weekly dosing.[11] Treatment switching and subsequent dose escalation indicate poor management of UC.

Our analysis found that mean all-cause healthcare costs for the 12 months post-index were greater for dose escalating than non-escalating patients (£14,596 vs. £13,351). The same pattern was seen for UC-related healthcare costs. Patients with prior infliximab therapy who dose escalated incurred lower costs than non-escalating patients due to fewer inpatient visits to obtain their treatment.

Prescriptions were the main driver of cost for UC patients and accounted for 96.49% of UC-related healthcare costs (£11,090 in all patients; £11,423 in the combined dose escalated cohort and £10,834 in the non-escalated cohort).

A limitation of this study is that the HTI database only includes hospital-based healthcare activities and does not capture data outside of this setting; thus costs are likely to be under-reported. Additionally, UC-related healthcare costs may be underestimated in the study as most outpatient and A&E visit costs were not attributable to a diagnosis as this not a requirement for reimbursement. Further studies should explore the long-term pathway of care and how dose escalation may still occur.

### Conclusions

The proportion of UC patients who dose escalate adalimumab in maintenance treatment is higher than previously reported in clinical studies. Patients who dose escalate also incur greater costs than non-escalators and those switching from another TNF- α inhibitor to adalimumab are also likely to escalate their dosage. Dose escalation is associated with an increase in healthcare costs and indicates inadequate management of UC that has not been considered previously.

## Supporting Information

S1 Fig A graphic illustration of the change in dispensing frequency. (TIF)

S2 Fig A graphic illustration of the change in dispensing quantity. (TIF)
