# Supplementary figures and images for: Dose Escalation and Healthcare Resource Use among Ulcerative Colitis Patients Treated with Adalimumab in English Hospitals: An Analysis of Real-World Data
Source: PLoS One. 2016 Feb 26;11(2):e0149692. doi: 10.1371/journal.pone.0149692 (PMC4768958; doi:10.1371/journal.pone.0149692)

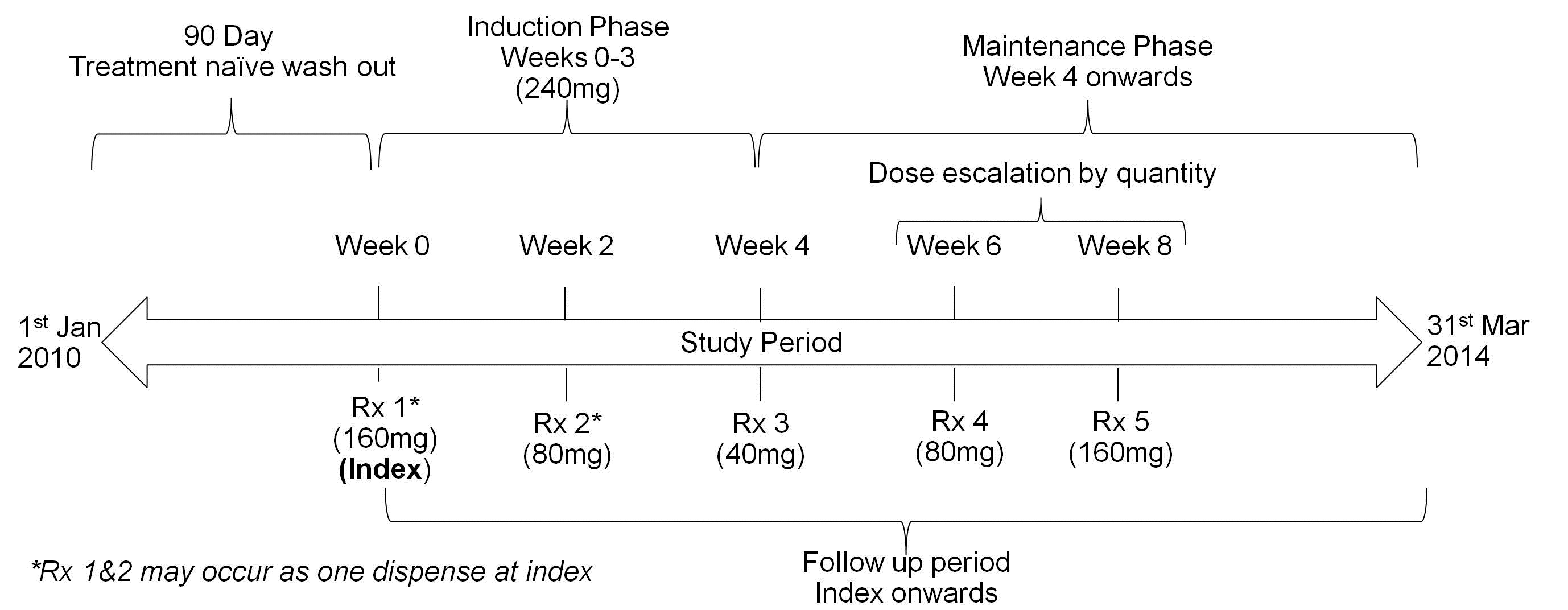

Supplement: S1 Fig — (TIF) [file pone.0149692.s001.tif]

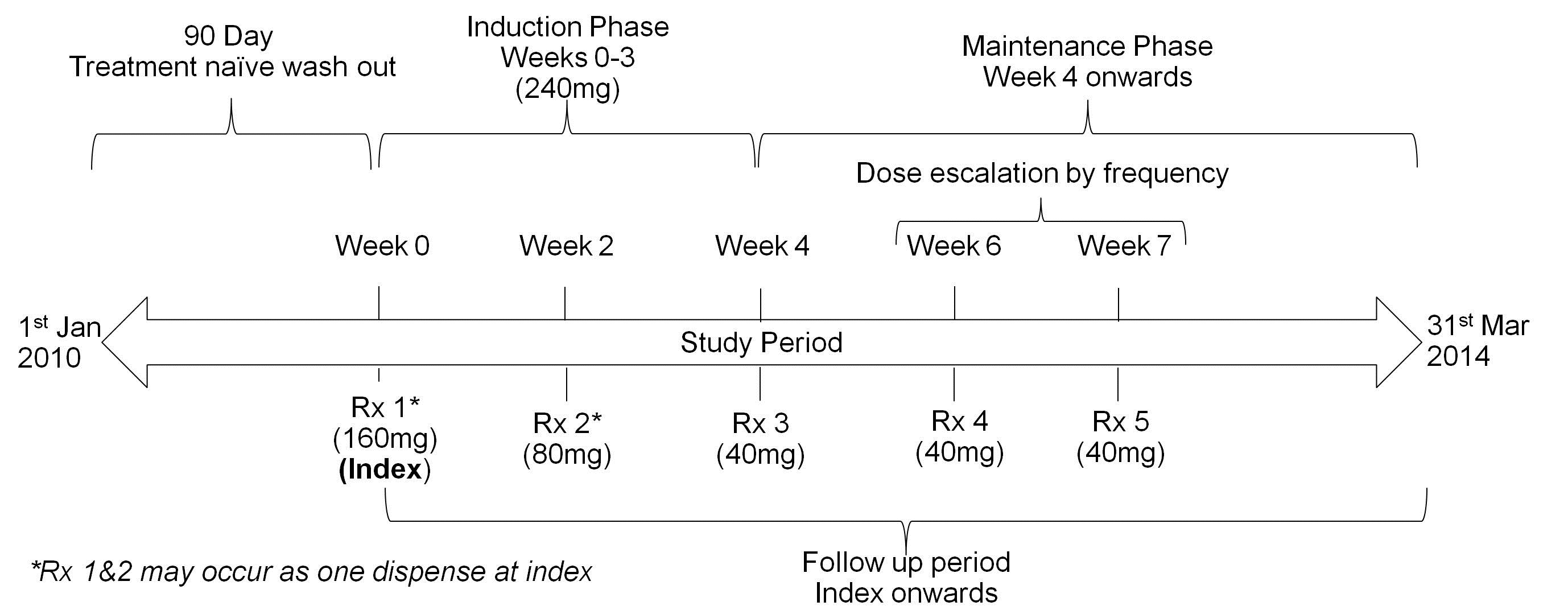

Supplement: S2 Fig — (TIF) [file pone.0149692.s002.tif]
